# Hyperglycemia Induces Endoplasmic Reticulum Stress in Atrial Cardiomyocytes, and Mitofusin-2 Downregulation Prevents Mitochondrial Dysfunction and Subsequent Cell Death

**DOI:** 10.1155/2020/6569728

**Published:** 2020-10-22

**Authors:** Ming Yuan, Mengqi Gong, Zhiwei Zhang, Lei Meng, Gary Tse, Yungang Zhao, Qiankun Bao, Yue Zhang, Meng Yuan, Xing Liu, Guangping Li, Tong Liu

**Affiliations:** ^1^Tianjin Key Laboratory of Ionic-Molecular Function of Cardiovascular Disease, Department of Cardiology, Tianjin Institute of Cardiology, Second Hospital of Tianjin Medical University, Tianjin 300211, China; ^2^Department of Cardiology, The First Affiliated Hospital of Anhui Medical University, Hefei, China; ^3^Tianjin Key Laboratory of Exercise Physiology and Sports Medicine, Department of Health & Exercise Science, Tianjin University of Sport, Tianjin 300381, China

## Abstract

Mitochondrial oxidative stress and dysfunction play an important role of atrial remodeling and atrial fibrillation (AF) in diabetes mellitus. Endoplasmic reticulum (ER) stress has been linked to both physiological and pathological states including diabetes. The aim of this project is to explore the roles of ER stress in hyperglycemia-induced mitochondrial dysfunction and cell death of atrial cardiomyocytes. High glucose upregulated ER stress, mitochondrial oxidative stress, and mitochondria-associated ER membrane (MAM)- enriched proteins (such as glucose-regulated protein 75 (GRP75) and mitofusin-2 (Mfn2)) of primary cardiomyocytes *in vitro*. Sodium phenylbutyrate (4-PBA) prevented the above changes. Silencing of Mfn2 in HL-1 cells decreased the Ca^2+^ transfer from ER to mitochondria under ER stress conditions, which were induced by the ER stress agonist, tunicamycin (TM). Electron microscopy data suggested that Mfn2 siRNA significantly disrupted ER-mitochondria tethering in ER stress-injured HL-1 cells. Mfn2 silencing attenuated mitochondrial oxidative stress and Ca^2+^ overload, increased mitochondrial membrane potential and mitochondrial oxygen consumption, and protected cells from TM-induced apoptosis. In summary, Mfn2 plays an important role in high glucose-induced ER stress in atrial cardiomyocytes, and Mfn2 silencing prevents mitochondrial Ca^2+^ overload-mediated mitochondrial dysfunction, thereby decreasing ER stress-mediated cardiomyocyte cell death.

## 1. Introduction

Atrial fibrillation (AF) is the most common sustained cardiac arrhythmia in humans and is associated with a fivefold increase in the risk of stroke. Numerous risk factors have been shown to promote AF, among which is diabetes mellitus (DM) [1, 2]. The growing recognition that diabetes and AF are closely related has stimulated interest in unraveling their relationship through mechanistic studies. In recent years, numerous studies have implicated that oxidative stress are interrelated pathways that promote atrial electrical and structural remodeling, leading to atrial ectopy and interstitial fibrosis [3, 4]. The ensuing mitochondrial dysfunction and DNA damage are key to the progression of different cardiovascular diseases, including diabetic cardiomyopathy and AF.

The endoplasmic reticulum (ER) is a specialized organelle that synthesizes, folds, and transports proteins in eukaryotic cells. Disruption of ER homeostasis leads to an accumulation of unfolded/misfolded proteins and ER stress. ER stress has been associated with both physiological and pathological conditions in the cardiovascular system including myocardial infarction, heart failure, hypoxia/reoxygenation injury, and dilated cardiomyopathy [5]. It is increasingly recognized that ER stress and unfolded protein response (UPR) pathways contribute to the pathogenesis of metabolic diseases such as diabetes [6]. For instance, our previous work has shown elevated levels of ER stress markers in DM patients with confirmed AF. However, the precise mechanism of how ER stress causes atrial electrical and structural remodeling is unclear. Questions remain as to whether ER stress contributes to mitochondrial oxidative stress and ROS production in the context of diabetes.

Mitochondria and ER are interconnected organelles, and the contact points through which ER communicates with mitochondria are termed mitochondria-associated ER membranes (MAMs) [7]. Several studies have identified several proteins enriched at the ER-mitochondria interface, one of them has identified a macromolecular complex composed of inositol 1,4,5-trisphosphate (IP3) receptors1 (IP_3_R1), glucose-regulated protein 75 (GRP75), and voltage-dependent anion channel (VDAC1) that regulates direct Ca^2+^ transfer from ER to mitochondria [8]. Recently, several MAM-enriched proteins were detected, including Mfn2, PACS2, and *σ*-1 receptor [9–11]. Mitofusin (Mfn) is a mitochondrial dynamin-related GTPase involved in mitochondrial Ca^2+^ regulation which includes Mfn1 and 2. In particular, Mfn2 is a critical component that participates in the fusion of the outer mitochondrial membranes of two adjacent mitochondria [12]. Mfn1 is exclusively localized to the mitochondrial outer membrane (OMM), while Mfn2 is found in both the OMM and the ER membrane [9, 13]. Tethers are composed of Mfn1 and Mfn2 proteins structurally attaching mitochondria to the ER; Mfn tethers mitochondria from the point source of ER Ca^2+^ release (via IP_3_Rs), termed “Ca^2+^ release microdomains.” This strategic positioning enables highly efficient, local mitochondrial Ca^2+^ uptake via the mitochondrial Ca^2+^ uniporter (MCU) [14, 15].

In the present study, we examined the effects of ER stress inhibitors (4-PBA) and ER stress agonist tunicamycin (TM) on mitochondrial Ca^2+^ handling and oxidative stress. Considering the role of Mfn2 at the ER-mitochondrial interface in atrial cardiomyocytes, we demonstrate that Mfn2 silencing prevents mitochondrial Ca^2+^ overload-mediated mitochondrial dysfunction, thereby decreasing ER stress-mediated cell death of cardiomyocytes.

## 2. Methods

Detailed methods are available in the Supplementary Material.

### 2.1. Isolation and Culture of Primary Cardiomyocytes

Primary atrial myocytes were separated from 2~3-day-old neonatal Sprague-Dawley (SD) rats, as our study described previously [16]. Briefly, atrial tissue was finely minced to 1 mm^3^ in cold phosphate-buffered saline (PBS) solution without Ca^2+^ and Mg^2+^ and mechanically digested with trypsin-type II collagenase buffer (0.125%) at 37°C until the tissue is invisible. The cell suspension was plated in a cell culture flask for 2 h to separate the fibroblasts and cardiomyocytes. Supernatants containing cardiomyocytes were plated in a new cell culture flask. The atrial myocytes were cultured in Dulbecco's Modified Eagle's Medium (DMEM, Gibco) containing 1.0 g/L D-glucose, 10% fetal bovine serum (FBS, Gibco), 100 IU/mL penicillin-streptomycin (Gibco), and 0.1 mM bromodeoxyuridine (BrdU) to inhibit proliferation of fibroblasts and help purify cardiac myocytes. The cells were randomly divided into four groups: control (normal glucose, NG), high glucose (HG), HG+sodium phenylbutyrate (4-PBA, 500 nM), and NG+4-PBA groups. The cells were pretreated with vehicle or 4-PBA for 1 hour and then stimulated with 25 mM glucose for 48 hours.

### 2.2. Cell Culture, siRNA Transfection, and Treatment

The HL-1 cell line was used for in vitro studies and maintained in Claycomb media (Sigma-Aldrich, MO), containing 10% fetal bovine serum (FBS, Gibco, MA, USA), 100 g/mL penicillin/streptomycin, 0.1 mM norepinephrine, and 2 mM L-glutamine. HL-1 cells were seeded into six-well plates, when cells reached 70-80% confluence, they were transfected with control siRNA (sc-37007) or Mfn2 siRNA (sc-60077, both Santa Cruz Biotechnology, CA) by using a Lipofectamine 3000 transfection reagent (Thermo Fisher Scientific, MA) for 24 h. Then, HL-1 cells were treated with 200 ng/mL TM (Solarbio Life Sciences, Beijing) for the next 24 h. DS16570511 (MedChemExpress, USA) is a cell-permeable inhibitor of the mitochondrial calcium uniporter, which blocks the MCU-dependent increase of Ca^2+^ influx. The cells were pretreated with DS16570511 (100 *μ*M) for 1 hour and then stimulated with TM for 24 hours. After TM exposure, cells were washed with PBS or HBSS for other measurements.

### 2.3. RNA Extraction and RT-qPCR

Total RNA was extracted from 1 × 10^6^ cells/mL using the Eastep Super Total RNA Extraction Kit (Promega) according to the manufacturer's protocol. RNA concentration and quality were immediately determined using a NanoDrop 2000 (Thermo Fisher Scientific). Total RNA (2 *μ*g) was used as a template to synthesize cDNA using the cDNA Synthesis Kit (TIANGEN, Guangzhou) according to the manufacturer's instructions. Reactions were carried out using a StepOnePlus Real-Time PCR System (Applied Biosystems, USA). The qPCR was performed using the following rat primers: GRP78 sense (5′-GGATAAGAGAGAGGGAGAGAAGA-3′) and antisense (5′-CCCAGATGAGTGTCTCCATTAG-3′), CHOP sense (5′-GAGAGTGTTCCAGAAGGAAGTG-3′) and antisense (5′-ACTGTCTCAAAGGCGAAAGG-3′), and GAPDH sense (5′-TCTCCCTCACAATTTCCATCC-3′) and antisense (5′-GGGTGCAGCGAACTTTATTG-3′), and the following mouse primers: Mfn2 sense (5′-TTGCTCCAGGTGGTTAAAGG-3′) and antisense (5′-CCTCAGTGCTAGGTCAGATAGA-3′) and GAPDH sense (5′-AACAGCAACTCCCACTCTTC-3′) and antisense (5′-CCTGTTGCTG-TAGCCGTATT-3′). Gene expression was normalized to the mRNA expression level of GAPDH as an internal control, and fold changes in expression were calculated between treatment and untreated control groups.

### 2.4. Mitochondrial Membrane Potential/ROS Assessment

HL-1 cells were loaded in 15 mm glass bottom dishes, then stained with 10 *μ*M 2′,7′-dichlorofluorescin diacetate (DCFH-DA, Sigma-Aldrich) for 20 min to quantify ROS and JC-1 (Beyotime Biotech, Haimen, China) staining solution for 20 min to quantify changes in mitochondrial membrane potential (*Δψ*m). Cells were imaged in a widefield fluorescence microscope (Olympus, IX81, Tokyo), and changes in intensity means were quantified using flow cytometry.

### 2.5. Assessment of Intracellular and Mitochondrial Calcium

The intracellular calcium level was measured by detecting the fluorescence of cells treated with a calcium-sensitive indicator Fluo-4 AM (Thermo Fisher Scientific, MA). The primary cardiomyocytes and HL-1 cells were treated with 5 *μ*M Fluo-4 AM and 0.02% (*W*/*V*) Pluronic F-127 in culture medium for 20 min in a CO_2_ incubator. After washing with HBSS (without Ca^2+^ and Mg^2+^), the cells were incubated without Fluo-4 AM for 15 min to ensure complete dye deesterification. Fluorescence was detected using an Olympus FV1000 LSCM confocal microscope; the signal was detected at an excitation and emission wavelength of 494 and 516 nm, respectively.

Mitochondrial Ca^2+^ content in primary cardiomyocytes and HL-1 cells was measured as described previously, with minor modifications [17]. Cells were loaded with 5 *μ*mol/L Rhod2 AM (Thermo Fisher Scientific) and 0.02% Pluronic F-127 for 30 min at room temperature and superfused with HBSS for 30 min to wash away mitochondrial AM esters. Rhod2 AM fluorescence (530 nm excitation; 576 nm emission) were imaged at room temperature using a fluorescence microscope. Changes in intensity means in both Fluo-4 AM and Rhod2 AM were quantified using flow cytometry.

### 2.6. Mitochondrial Respiration Assays

The HL-1 cell mitochondrial oxygen consumption rate (OCR) was measured using the Seahorse Bioscience XF24 Extracellular Flux Analyzer (Seahorse Bioscience) according to the manufacturer's protocol. Cells were grown on a plastic 24-well plate; after being transfected with siRNA and treated with TM, ATP production, maximal respiration, and nonmitochondrial respiration were assessed by treating cells with 10 *μ*M oligomycin, 10 *μ*M FCCP, and 10 *μ*M antimycin A (AA) + Rotenone mixture, respectively.

### 2.7. Transmission Electron Microscopy

Mitochondrial and ER morphology and associations were assessed by transmission electron microscopy (TEM). HL-1 cells were collected and fixed with 2.5% glutaraldehyde in 0.2 M phosphate buffer (pH 7.3–7.4) at 4°C overnight. Samples were then dehydrated by a series of exposures to ethanol, infiltrated with 100% propylene oxide, and embedded in an epoxy resin for 24 h. Ultrathin sections (70 nm) were prepared, and ER-mitochondria were imaged on a JOEL JEM1230 (JOEL, Tokyo, Japan). The operator and analyzer were blinded to the experimental condition and quantified mitochondrial and ER morphology and contact length using ImageJ.

### 2.8. Flow Cytometry of Cell Apoptosis

The mode of cell apoptosis was evaluated by flow cytometry using Annexin V-fluorescein isothiocyanate (FITC)/propidium iodide (PI) (Kaiji, Nanjing, China) staining. After the incubation period, 500 *μ*L of untreated and TM-treated cells was transferred to separate tubes and 5 *μ*L Annexin V-FITC and 5 *μ*L PI were added to each tube. The tubes were incubated at room temperature for 15 min in the dark, then analyzed with flow cytometry (FACSVerse, BD, USA) within 1 h. Cell apoptosis was analyzed using a flow cytometer.

### 2.9. Western Blot Analysis

Cultured cells were lysed in RIPA buffer plus 1 mmol/L phenylmethylsulfonyl fluoride (PMSF, Solarbio Life Sciences, Beijing) for 30 min at 4°C. Proteins (60 *μ*g) were separated by SDS-PAGE and transferred to PVDF membranes, blocked with TBST containing 5% nonfat milk and incubated overnight at 4°C with the following antibodies: anti-CHOP (#2895), anti-caspase9 (#9504S), anti-caspase3 (#9662S), anti-cleaved-caspase9 (#9509S), anti-cleaved-caspase3 (Asp175) (#9661S), and anti-MCU (D2Z3B) (#14997S) from Cell Signaling Technology; anti-GRP78 (ab21685), anti-Mfn2 (ab56889), anti-VDAC1 (ab15895), anti-Bax (ab32503), anti-Bcl-2 (ab59348), and anti-Mn-SOD (ab13533) from Abcam; and anti-*β*-actin antibody as a loading control from Proteintech. Proteins were detected by using the ECL method, and the reactions were visualized using the Tanon 5200 Multi Chemiluminescent Imaging System (Tanon Science & Technology Co., Ltd., Shanghai, China).

### 2.10. Statistical Analysis

Data were expressed as mean ± SD. Student's *t* test was used for the comparisons between 2 groups. ANOVA was used to make comparisons between multiple groups. *p* < 0.05 was considered statistically significant.

## 3. Results

### 3.1. Exposure to High Glucose Upregulates ER Stress Markers and Ca^2+^ Levels of Primary Cardiomyocytes *In Vitro*

**Figure 1:**
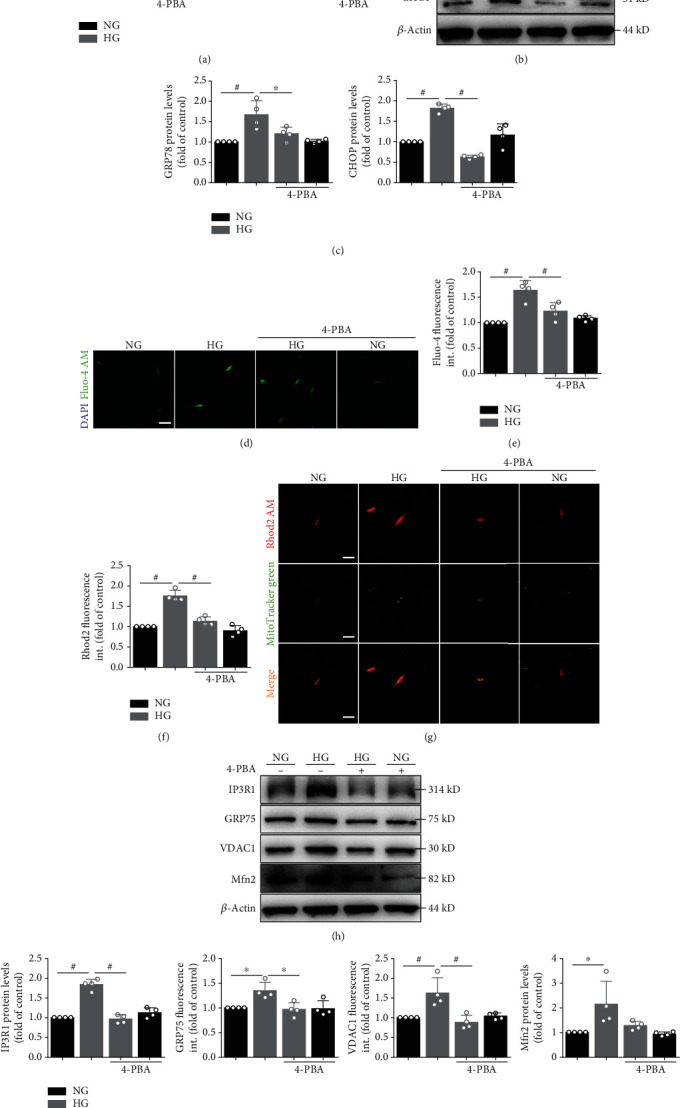
High glucose induces ER stress and upregulates intracellular and mitochondrial Ca^2+^ levels. (a) Primary atrial myocytes under normal glucose (NG) or high glucose (HG) with or without 4-PBA for 24 hours. Quantification of GRP78 and CHOP mRNA expression. (b) Primary atrial myocytes under NG or HG with or without 4-PBA for 48 hours. GRP78 and CHOP protein level in atrial myocytes detected by Western blot analysis. (c) Quantification of GRP78 and CHOP protein level in (b). (d) Representative confocal microscopy images of fluorescence staining for Fluo-4 AM and DAPI. Scale bar, 20 *μ*m. (e) Quantification of Fluo-4 AM fluorescence intensity in (d). (f) Representative confocal microscopy images of fluorescence staining for Rhod2 AM and MitoTracker Green. Scale bar, 20 *μ*m. (g) Quantification of Rhod2 AM fluorescence intensity in (f). (h) MAM proteins such as IP_3_R1, GRP75, VDAC1, and Mfn2 protein level in atrial myocytes detected by Western blot analysis. (i) Quantification of IP3R1, GRP75, VDAC1, and Mfn2 protein level in (h). Data are mean ± SEM, *n* = 4 independent experiments, ^∗^*p* < 0.05, ^#^*p* < 0.01, ANOVA with Bonferroni posttest.

To determine the direct effect of high glucose on ER stress activation, primary cardiomyocytes were cultured at normal or high glucose conditions. Elevated glucose environment produced a significant increase in the levels of glucose-regulated protein 78 (GRP78) and C/EBP-homologous protein (CHOP) mRNA expression (Figure 1(a)). Similar results were found in the protein expression, where a significant increase in the protein levels of GRP78 and CHOP in the group of HG (Figures 1(b) and 1(c)). The ER stress levels were markedly decreased in the HG+4-PBA (4-PBA, a classical ER stress inhibitor) group (*p* < 0.01) compared with the HG group (Figures 1(a)–1(c)).

Triggered activity caused by delayed afterdepolarizations (DADs) is typically needed for the initiation of AF. DADs arise from an increased Ca^2+^ leak from the ER. To further find the ER-mitochondria Ca^2+^ transfer, the intracellular Ca^2+^ level was measured by Fluo-4 staining (Figures 1(d) and 1(e)) and the mitochondria Ca^2+^ level (Figures 1(f) and 1(g)) was measured by Rhod2 staining. The intracellular and mitochondria Ca^2+^ increased obviously after 48 h of high glucose stimulation. In addition, 4-PBA could decrease the high glucose-induced Ca^2+^ overload (Figures 1(d)–1(g)).

**Figure 2:**
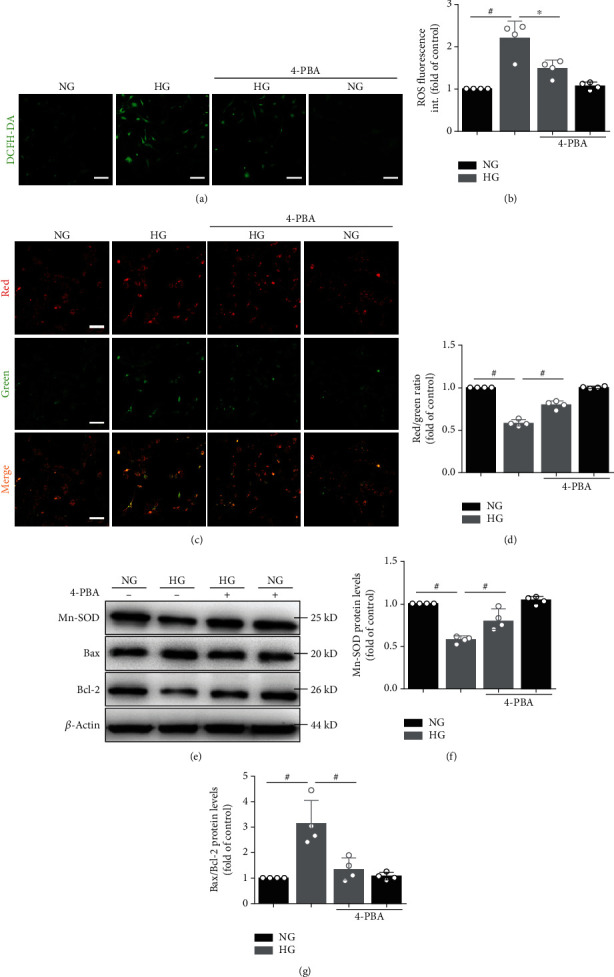
High glucose induces mitochondrial oxidative stress and induced apoptosis. (a) Confocal microscopy detected primary atrial myocytes that stained with DCFH-DA. (b) Quantification of ROS by DCFH-DA intensity in (a). Data represent the mean ± SEM (*n* = 4 independent experiments). (c, d) JC-1 staining. The ratio of red/green fluorescence reflects changes in the *Δψ*m of primary atrial myocytes. Data represent the mean ± SEM (*n* = 4 independent experiments). Scale bar, 100 *μ*m. (e) Mn-SOD, Bax, and Bcl-2 protein level in atrial myocytes detected by Western blot analysis. (f, g) Quantification of Mn-SOD and Bax/Bcl-2 protein level in (e). Data are mean ± SEM, *n* = 4 independent experiments, ^∗^*p* < 0.05, ^#^*p* < 0.01, ANOVA with Bonferroni posttest.

We also measured MAM-enriched proteins (Figures 1(h) and 1(i)), mitochondrial oxidative stress (Figures 2(a) and 2(b) and 2(e) and 2(f)), mitochondrial membrane potential (*Δψ*m, Figures 2(c) and 2(d)), and apoptosis-related (Figures 2(e)–2(g)) protein levels. Western blot analysis showed that the protein levels of IP_3_R1, GRP75, VDAC1, Mfn2, and Bax in primary cardiomyocyte cells were significantly higher, whereas the levels of Mn-SOD and Bcl-2 were lower in the HG group than in the NG group. Intracellular ROS levels were significantly elevated, and *Δψ*m were significantly reduced in the HG group. However, preincubation of myocytes with 4-PBA inhibitors partly prevented the effect of high glucose on protein production. The preincubation itself did not significantly alter the control protein levels (Figures 1(h) and 1(i) and 2(a)–2(g)).

### 3.2. Mfn2 Removal from the MAMs Changes Atrial Myocyte Ultrastructure and Decreases the Interactions between ER-Mitochondria

**Figure 3:**
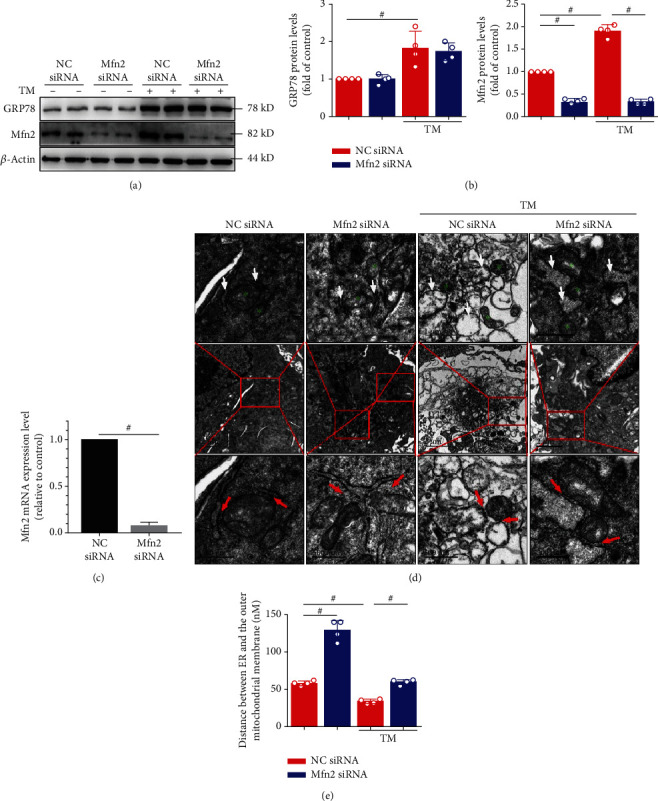
Mfn2 siRNA disrupts endoplasmic reticulum- (ER-) mitochondria tethering in tunicamycin- (TM-) injured HL-1 cells. (a) Changes in GRP78 and Mfn2 levels induced by TM were examined using Western blot analysis. The silencing efficiency of Mfn siRNA also shown in (a). (b) Quantification of GRP78 and Mfn2 protein level in (a). (c) Quantification of Mfn2 mRNA expression. (d) Ultrastructural changes in HL-1 cells (green stars depicting the mitochondria, white arrowheads depicting the endoplasmic reticulum). Transmission electron micrograph images (red arrowheads depicting the ER-mitochondria contacts) and measurements of the ER-mitochondria distance (e). Scale bar, top 1 *μ*m, middle 2 *μ*m, and bottom 500 nm. Data are mean ± SEM, *n* = 4 independent experiments, ^#^*p* < 0.01, ANOVA with Bonferroni posttest.

ER stress levels were markedly increased in the HG group (both in primary cardiomyocytes and HL-1 cells (Supplementary Figure 1); to examine the role of ER stress in AF progression, next, we applied TM, a classical ER stress inducer. Mfn2 is involved in tethering ER to mitochondria and regulating ER-mitochondria cross talk. GRP78 and Mfn2 protein expression was substantially increased after TM was compared with the control group (Figure 3(a)). Furthermore, Mfn2 mRNA levels and protein expression were downregulated by the corresponding siRNA (Figures 3(a) and 3(c)). The ultrastructure of HL-1 cells is illustrated in Figure 3(d). In the negative control (NC) siRNA group, regular ER organization and uniformly sized mitochondria between ERs were observed. However, the NC siRNA+TM group showed swelling mitochondria that were accompanied by dilatation and vesiculation of the endoplasmic reticulum. In the Mfn2 siRNA-treated TM group, partial dilatation and vesiculation of endoplasmic reticulum and slight swelling of mitochondria were observed.

To investigate the interactions between the ER and mitochondria, we analyzed critical parameters related to their functional interactions and the distance between the ER and the mitochondria. First, we attempted to measure ER-mitochondria distance with immunofluorescence assays (ER-Tracker Red marks ER and MitoTracker Green marks mitochondria). However, the resolution of the confocal microscope is too low to achieve accurate measurement (Supplementary Figure 2). As described previously [18], we used ImageJ software to analyze the distance between ER and the outer mitochondrial membrane, in the transmission electron micrographs (Figures 3(d) and 3(e)). Our results showed that the mean distance between ER and the OMM was decreased in the TM+NC siRNA group. The ER-mitochondria distance at these sites was increased in the Mfn2 siRNA-treated TM group (Figures 3(d) and 3(e)). Our results suggested that Mfn2 siRNA markedly disrupted ER-mitochondria tethering in ER stress-injured HL-1 cells.

### 3.3. Mfn2 Controls the Ca^2+^ Transfer from ER to Mitochondria

**Figure 4:**
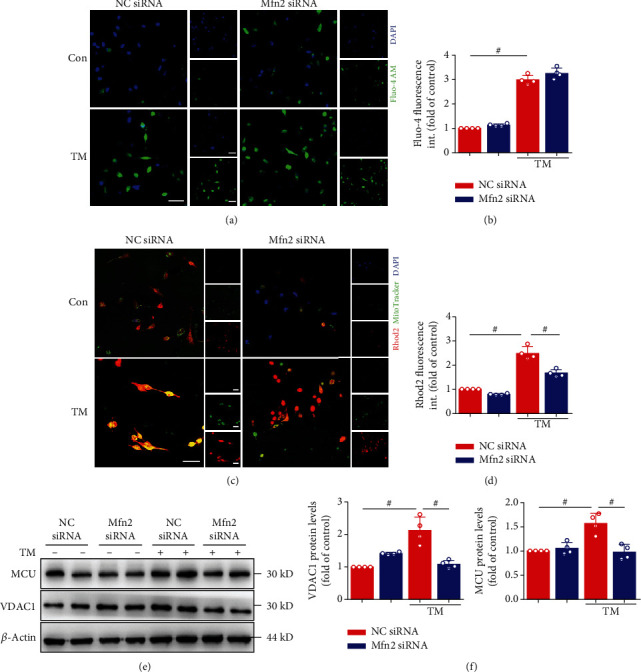
Mfn2 siRNA inhibited mitochondrial Ca^2+^ overload during ER stress induced by TM. (a) Representative confocal microscopy images of fluorescence staining for Fluo-4 AM and DAPI. Scale bar, 20 *μ*m. (b) Quantification of Fluo-4 AM fluorescence intensity in (a). (c) Representative confocal microscopy images of fluorescence staining for Rhod2 AM and MitoTracker Green. Scale bar, 20 *μ*m. (d) Quantification of Rhod2 AM fluorescence intensity in (c). (e) Mitochondria Ca^2+^ proteins such as VDAC1 and MCU protein level in atrial myocytes detected by Western blot analysis. (f) Quantification of VDAC1 and MCU protein level in (e). Data are mean ± SEM, *n* = 4 independent experiments, ^#^*p* < 0.01, ANOVA with Bonferroni posttest.

Given that the IP_3_R1/Grp75/VDAC1 complex has been shown to directly control the Ca^2+^ transfer from ER to mitochondria [8], we hypothesized that Mfn2 plays a part in this Ca^2+^ exchange between the two organelles. Assessment of intracellular and specific mitochondrial Ca^2+^ loading with Fluo-4 and Rhod2 was performed on HL-1 cells after TM stimulation, with the confocal microscope proving the induced Ca^2+^ release from ER stores to cytoplasm and mitochondria (Figures 4(a)–4(d)). In HL-1cells, TM rapidly increased mitochondrial Ca^2+^ levels, reflecting IP_3_R-mediated Ca^2+^ transfer from ER to mitochondria. In the meanwhile, the protein expression of VDAC1 and MCU, which are located in the OMM and the inner mitochondrial membrane (IMM), respectively, was detected. As shown in Figures 4(e) and 4(f), TM increased VDAC1 and MCU protein expression. Downregulation of Mfn2 expression by specific siRNA led to a significant reduction of the TM-stimulated increase of mitochondrial Ca^2+^ relative to the TM group. These findings suggested that in cells lacking Mfn2, the distance between ER and mitochondria was increased, Ca^2+^ flow from ER to mitochondria was decreased, and mitochondrial Ca^2+^ uptake was impaired.

### 3.4. Silencing the Mfn2 Decreases ROS and Improves Mitochondrial Function of Atrial Myocytes

**Figure 5:**
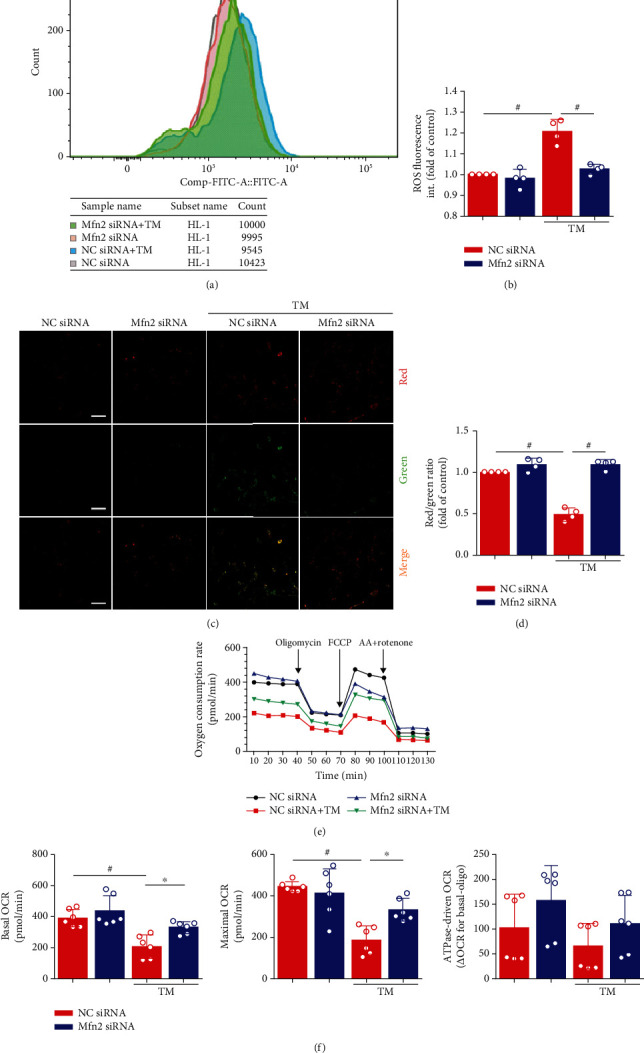
Decreasing ER-mitochondria interactions by genetic downregulation of mitofusin-2 (Mfn2) protects HL-1 cells from mitochondria dysfunction. (a) Flow cytometry detected HL-1 cells that stained with DCFH-DA. (b) Quantification of ROS by DCFH-DA intensity in (a). Data represent the mean ± SEM (*n* = 4 independent experiments). (c, d) JC-1 staining. The ratio of red/green fluorescence reflects changes in the mitochondrial membrane potential of HL-1 cells. Data represent the mean ± SEM (*n* = 4 independent experiments). Scale bar, 100 *μ*m. (e, f) Analysis of oxygen consumption rate (OCR) from a Seahorse XF 24 Extracellular Flux Analyzer. Oligomycin inhibits ATP synthase, FCCP uncouples oxygen consumption from ATP production, and AA+Rotenone inhibits complexes I and III, respectively. Data represent the mean ± SEM (*n* = 6 independent experiments), ^#^*p* < 0.01.

We used DCFH-DA to detect changes in intracellular ROS levels. Intracellular ROS levels were significantly elevated after ER stress (Figure 5(a)). These results indicated that intracellular oxidative stress occurred in HL-1 cells subjected to ER stress and that mitochondria may play a significant role in this process. Interestingly, Mfn2 siRNA also decreased ER stress-induced intracellular ROS levels (Figures 5(a) and 5(b)).

Ca^2+^ flux from the ER and high levels of mitochondrial accumulation are associated with the effects of several apoptosis signals. Destruction of *Δψ*m is widely regarded as one of the earliest events in the process of apoptosis. The *Δψ*m was quantified using the JC-1 dye. If loss or collapse of *Δψ*m occurred, the ratio of red/green fluorescence was reduced. After ER stress, the dye remained mostly in the cytoplasm and showed green fluorescence (Figures 5(c) and 5(d)). In contrast, the dye in the Mfn2 siRNA-treated TM group seemed to accumulate within mitochondria and showed red fluorescence.

To assess whether this *in vitro* model of mitochondrial fragmentation is associated with mitochondrial dysfunction, respiration was assessed by Seahorse oximetry. TM treatment decreased both basal respiration (before oligomycin) and maximum respiratory capacity (after FCCP), and downregulation of Mfn2 expression improved the above changes (Figures 5(e) and 5(f)). The mitochondrial genome (mtDNA) quantification provides evidence that the above changes are not due to the changes in mitochondrial content (Supplementary Figure 3).

### 3.5. Downregulation of Mfn2 Protects HL-1 Cells from ER Stress-Induced Apoptosis

**Figure 6:**
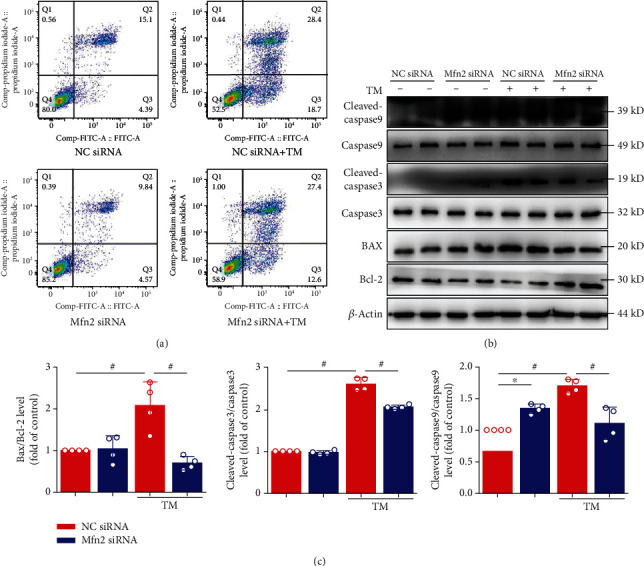
Mfn2 siRNA inhibited endoplasmic reticulum- (ER-) mitochondria-dependent apoptosis. (a) Cell apoptosis was evaluated by flow cytometry using Annexin V-fluorescein isothiocyanate (FITC)/propidium iodide (PI) staining. (b) HL-1 cells under TM stimulated for 24 hours. Mfn siRNA inhibited the Bax/Bcl-2 ratio and inhibited caspase3 and caspase9 activation (c). Data are mean ± SEM, *n* = 4 independent experiments, ^∗^*p* < 0.05, ^#^*p* < 0.01, ANOVA with Bonferroni posttest.

Flow cytometry was conducted to detect cell apoptosis by Annexin V-FITC/PI staining (Figure 6(a)). ER stress caused a significant increase in cell apoptosis, and Mfn2 siRNA administration markedly decreased TM-induced apoptosis. We also determined the antiapoptotic factor Bcl-2 expression and the Bax/Bcl-2, cleaved-caspase3/caspase3, and cleaved-caspase9/caspase9 ratio during the TM treatment. In the control group, there were relatively low levels of Bax and cleaved-caspase3/9 protein expressions. After TM procedures, the above protein levels were substantially increased in the HL-1 cells. Furthermore, the increased cell death observed after ER stress was significantly attenuated in the Mfn2 siRNA group (Figures 6(b) and 6(c)). We further showed that DS16570511 inhibits mitochondrial Ca^2+^ influx and prevents the TM-induced cell death in HL-1 cells (Supplementary Figure 4A-D). These results indicate that disruption of ER-mitochondria interaction though Mfn2 tethering prevents the Ca^2+^ transfer from ER to mitochondria during ER stress and attenuates mitochondrial Ca^2+^ loading and the subsequent cell apoptosis.

## 4. Discussion

The main findings of this study are that inhibition of Mfn2 attenuated mitochondrial oxidative stress and Ca^2+^ overload, increased mitochondrial membrane potential and mitochondrial oxygen consumption, and protected cells from ER stress-induced apoptosis.

**Figure 7:**
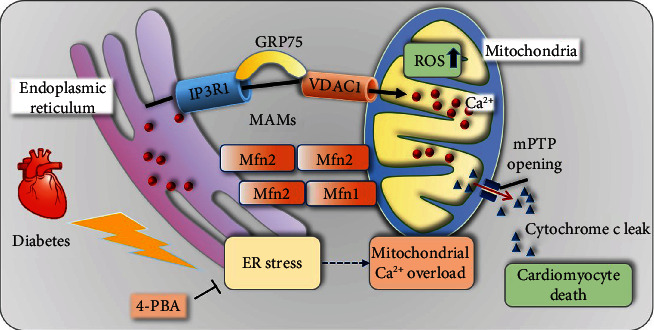
Central illustration. Mfn2 deficiency in this study protected cardiomyocytes against mitochondrial depolarization/reactive oxygen species (ROS) and programmed cell death, improving atrial remodeling.

Tunicamycin has previously been shown to initiate ER stress-increased protein levels of GRP78, CHOP, and the proapoptotic proteins caspase3 and caspase9 and the Bax/Bcl-2 ratio. Consistent with Mfn2 functioning as the ER-mitochondrial tether, ablation of Mfn2 in HL-1 cells increased the spatial separation between ER and mitochondria and decreased mitochondrial Ca^2+^ uptake after stimulation following the application of ER stress-inducing agents. Mfn2 deficiency in this study protected cardiomyocytes against mitochondrial depolarization/reactive oxygen species (ROS) and programmed cell death, improving atrial remodeling (Figure 7).

ER stress is a pathological condition that can be activated a wide range of cellular environments and events, including increased levels of protein synthesis, deficient autophagy, energy deprivation, limited or excess of nutrients, deregulated Ca^2+^ levels or redox homeostasis, inflammation, and hypoxia. The ER and the mitochondria in living cells are two essential organelles with distinct roles. The MAM fraction was first separated and characterized by Vance [19]. After this seminal observation, the evidence has been accruing that a specific interaction and cooperation between these compartments is required for cellular functions such as lipid metabolism, Ca^2+^ signaling, autophagy, cell survival, and death [20, 21]. Ca^2+^ is an important regulator of oxidative phosphorylation by stimulating rate-limiting enzymes of the Krebs cycle, increasing the availability of NADH and FADH2 for the electron transport chain [22, 23]. However, excessive transfer of ER calcium to mitochondria is a proapoptotic signal with important consequences for cell fate. Many stimuli induce Ca^2+^ release, but two channel families mainly control the ER Ca^2+^-release program: ryanodine receptors (RYRs) and IP_3_Rs [24, 25]. Triggered activity caused by delayed afterdepolarizations (DADs) is typically needed for the initiation of AF. DADs arise from increased Ca^2+^ leak from the ER via RYR2. Interestingly, IP_3_Rs appears to be enriched at MAMs [26], and its stabilization by Sig-1R ensures proper Ca^2+^ influx into mitochondria. RYRs and IP_3_Rs are regulated by multiple regulatory proteins and phosphorylation/dephosphorylation, some of them being further explicated. More recently, one group demonstrated that the MAM-enriched promyelocytic leukemia protein (PML) exerts an important Ca^2+^-dependent role in the autophagy, through the AMPK/mTOR/Ulk1 pathway [27]. Phosphatase and tensin homolog deleted on chromosome 10 (PTEN) was also found to be localized at the ER and MAMs, where it regulates Ca^2+^ transfer from ER to mitochondria in a protein phosphatase-dependent manner [28]. Taken together, these observations highlight the role of the dependence of ER Ca^2+^ release as a general mediator in many cell death or cell survival settings and enhance the importance of MAM in Ca^2+^ handling. In the case of ER stress and ER Ca^2+^ handling abnormalities, the Ca^2+^ in the ER not only is released into the cytoplasm but also flows into the mitochondria through MAM Ca^2+^ channels, which can affect mitochondria function and consequently energy metabolism [29]. Driven by the membrane potential of the inner mitochondrial membrane, Ca^2+^ can be transported to the mitochondrial matrix through the MCU which exists in the inner mitochondrial membrane. The main physiological role of MCU-mediated mitochondrial Ca^2+^ uptake is to balance energy supply and demand. Prior studies have shown that under pathological conditions such as ischemia/reperfusion injury, MCU-mediated mitochondrial Ca^2+^ influx plays an important role in inducing mPTP opening and cell death [30].

Another MAM protein, Mfn2, has also been proposed as a tethering complex that couples to mitochondrial Mfn1/2. In particular, Mfn2 is equally a critical component of the mitochondrial fusion or fission machinery. However, the role of Mfn2 at MAM has been debated as per several recent studies from different laboratories. Indeed, as early as 2008, de Brito and Scorrano indicated that Mfn2 is enriched at contact sites between the ER and mitochondria, regulating ER and mitochondria morphology and directly tethering the two organelles [9]. The distance between the ER and mitochondria increases in Mfn2-deficient cells, and this is expected to result in impaired mitochondrial Ca^2+^ uptake, further disturbing the mitochondrial Ca^2+^ homeostasis theory. The role of Mfn2 was supported by work conducted by other laboratories [31–33]. But recently, its theory was challenged by different experimental approaches. In contrast to previous studies, confocal microscopy analyses suggested that loss of Mfn2 increased, rather than decreased, the proximity between the organelles. They demonstrated that reduced Ca^2+^ transfer in Mfn2-knockout cells is the result of a lower expression of MCU and are independent of ER-mitochondria juxtapositions [18]. Recently, a critical reassessment of the role of Mfn2 in the ER-mitochondria connection was published, supporting previous results and identifying Mfn2 as a physical tether between the two organelles in multiple tissues [34]. Thus, whether Mfn2 is functionally involved via anti- or protethering in two organelles requires further work to be conducted.

### 4.1. Limitations of This Study

There are some limitations of this study. The model used for investigations was exclusively *in vitro*, which inevitably sacrifices the interconnectivity of cells of intact organs. Secondly, we did not examine the relationship between abnormalities at the cellular level to atrial remodeling and atrial fibrillation. Last but not the least, due to some experimental limitations, we were unable to quantify ER-to-mitochondria Ca^2+^ transfer in live cells.

## 5. Conclusions

Inhibition of Mfn2 attenuated mitochondrial oxidative stress and Ca^2+^ overload, increased mitochondrial membrane potential and mitochondrial oxygen consumption, and protected cells from ER stress-induced apoptosis.

## Data Availability

The data used to support the findings of this study are available from the corresponding author upon request.

## Supplementary Materials

Supplementary materials The supplementary material includes the graphical abstract of the manuscript.
