# Supplementary material for: Hyperglycemia Induces Endoplasmic Reticulum Stress in Atrial Cardiomyocytes, and Mitofusin-2 Downregulation Prevents Mitochondrial Dysfunction and Subsequent Cell Death
Source: Oxid Med Cell Longev. 2020 Oct 22;2020:6569728. doi: 10.1155/2020/6569728 (PMC7603626; doi:10.1155/2020/6569728)
Supplement: Supplementary materials — The supplementary material includes the graphical abstract of the manuscript. [file 6569728.f1.doc]

**Supplemental Material**

**Supplemental methods**

**DNA Isolation, Quantification and Real-time PCR**

Genomic DNA (gDNA) used in qPCR reactions was extracted from cells using a standard protocol. We use the Universal Genomic DNA Extraction Kit (Solarbio life Sciences, Beijing). The template concentration was determined using NanoDrop and adjusted to 3 ng/μl. To avoid errors arising from repeated freeze thaw cycles DNA samples were kept at 4 °C for the duration of study. The mitochondrial genome (mtDNA) and the nuclear DNA (nucDNA, B2M) content was assessed by absolute quantification using real time PCR. The reactions were performed in ABI 7500 Real Time PCR System using the following protocol: pre-incubation at 95 °C for 5 min (1 cycle); denaturation at 95 °C for 10 s, annealing and extension at 60 °C for 30 s (repeat steps for 40 cycles), melting at 95 °C for 5 s, 65 °C for 60 s, and 95 °C continues (1 cycle) and the last step, cooling at 40 °C for 30 s. The qPCR was performed using the following mouse primers: mtDNA sense (5′- CTAGAAACCCCGAAACCAAA-3′) and antisense (5′- CCAGCTATCACCAAGCTCGT-3′), B2M sense (5′- ATGGGAAGCCGAACATACTG-3′) and antisense (5′- CAGTCTCAGTGGGGGTGAAT-3′), mtDNA content calculated as the ratio of mitochondrial genome to nuclear genome, and fold changes in expression were calculated between treatment and untreated control groups.

**ER-Tracker and Mito-Tracker confocal imaging**

HL-1 cells were loaded in 15mm glass bottom dishes, then stained with 1 μM ER-Tracker Red (Kaiji, Nanjing, China) for 30 min to marks ER and 50nM Mito-Tracker Green (Kaiji, Nanjing, China) staining solution for 30 min to marks mitochondria. Cells were imaged in a widefield fluorescence microscope (Olympus, IX81, Tokyo).


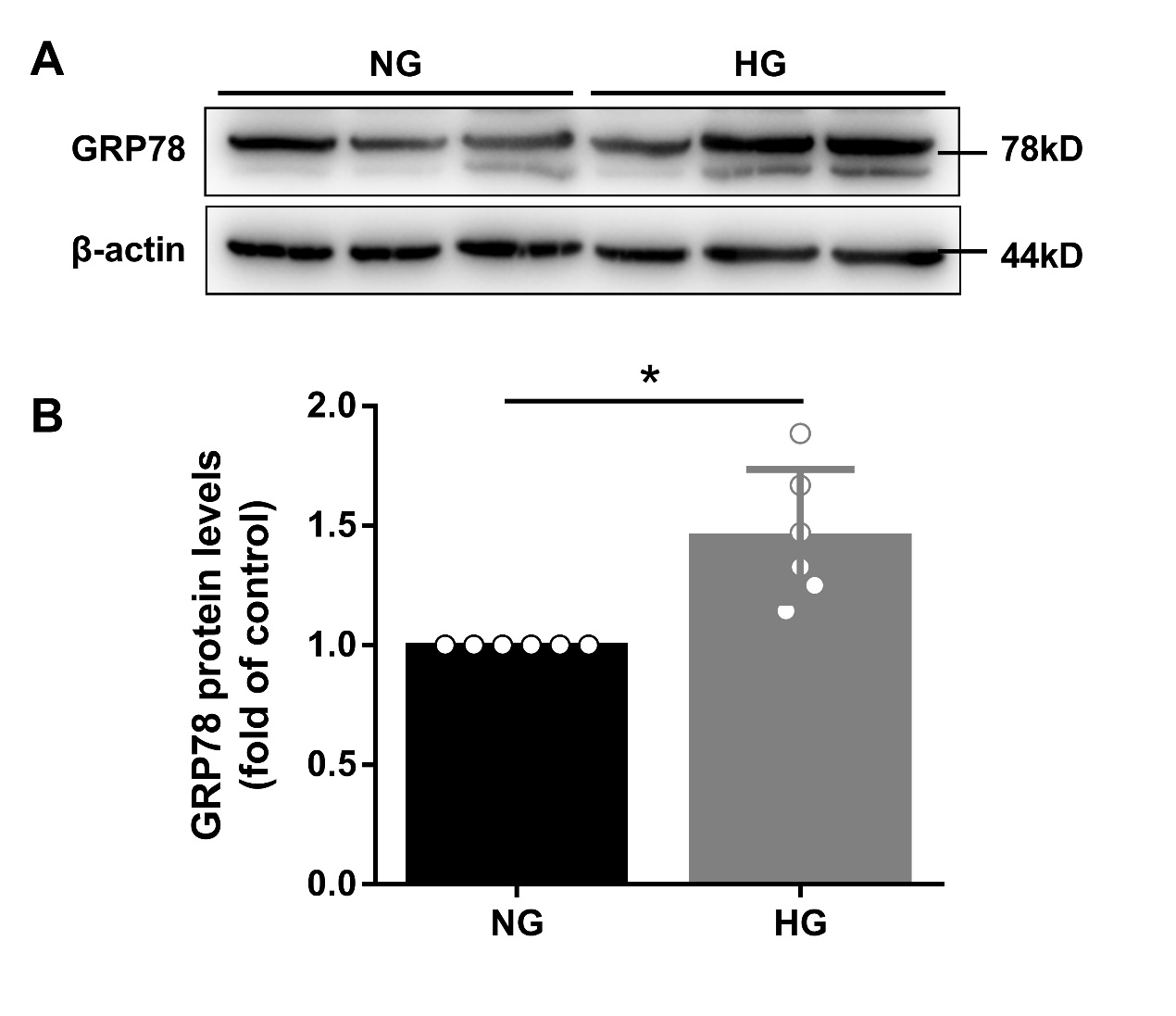


**Supplementary Figure 1.** High glucose induces ER stress in HL-1 cells. (A) HL-1 cells under normal glucose (NG) or high glucose (HG) for 48 hours. GRP78 protein level in HL-1 cells detected by western blot analysis. (B) Quantification of GRP78 protein level in (A).Values are the mean ± SEM, n = 6 independent experiments, two-tailed Student’s t-test.


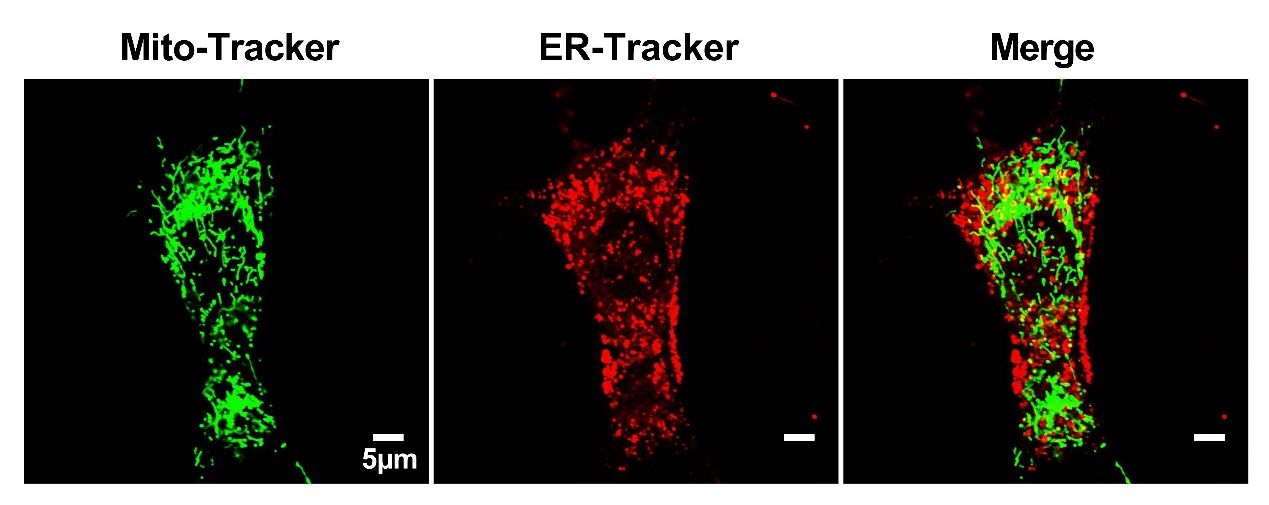


**Supplementary Figure 2.** Confocal images of HL-1 cells co-expressing an ER-targeted probe (ER-Tracker Red) and a mito-targeted probe (Mito-Tracker Green).

**
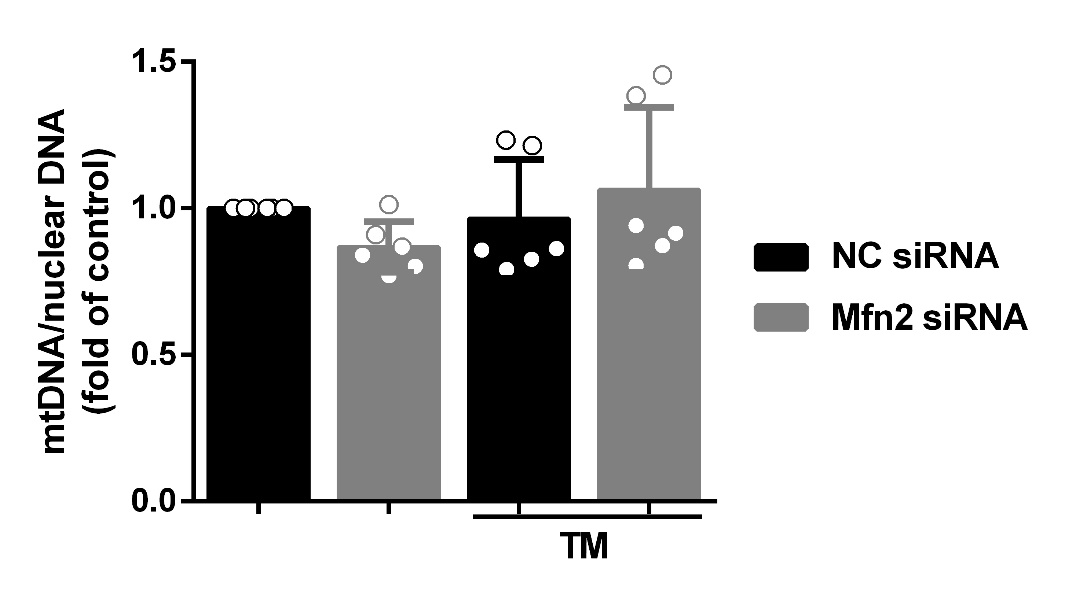
**

**Supplementary Figure 3.** Total DNA was extracted, quantified and diluted to 3 ng/μl, mtDNA content was quantified using absolute quantification with real-time qPCR. Data are shown as mean values ± SEM, mtDNA content (mito/nuclear) calculated as the ratio of mitochondrial genome to nuclear genome. n = 6 independent experiments, *p<0.05, # p<0.01, ANOVA with Bonferroni post-test.

**
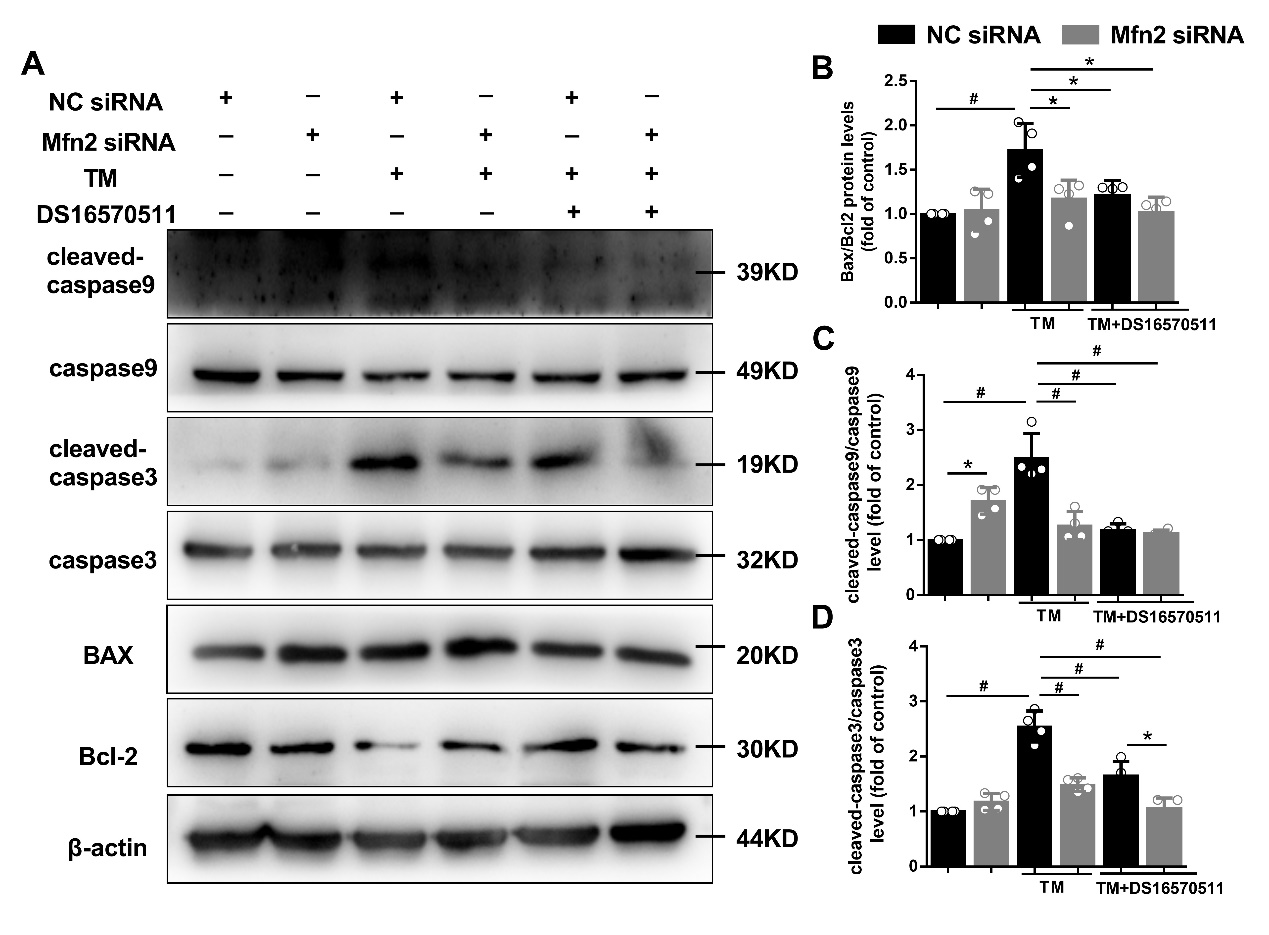
**

**Supplementary Figure 4.** DS16570511, an inhibitor of mitochondrial Ca2+ uptake, prevented the cell death. (A) HL-1 cells under DS16570511 and TM stimulated for 24 hours. DS16570511 and Mfn siRNA inhibited the Bax/Bcl-2 ratio, inhibited caspase3 and caspase9 activation (B-D). Data are mean ± SEM, n = 4 independent experiments, *p<0.05, # p<0.01, ANOVA with Bonferroni post-test.
